# An Evaluation of Treatment Outcomes and Associated Factors in Nasopharyngeal Cancer Patients at a Tertiary Cancer Center in the United Arab Emirates

**DOI:** 10.7759/cureus.54344

**Published:** 2024-02-17

**Authors:** Abdulrahman Bin Sumaida, Nandan M Shanbhag, Hussain A Aby Ali, Noor Jaafar, Khalifa AlKaabi, Khalid Balaraj

**Affiliations:** 1 Oncology/Radiation Oncology, Tawam Hospital, Al Ain, ARE; 2 Oncology/Palliative Care, Tawam Hospital, Al Ain, ARE; 3 College of Medicine and Health Sciences, United Arab Emirates University, Al Ain, ARE; 4 Radiology, Tawam Hospital, Al Ain, ARE; 5 Radiotherapy Technology, Tawam Hospital, Al Ain, ARE

**Keywords:** epstein barr virus (ebv), demographics, sib, radiotherapy, united arab emirate, nasopharyngeal neoplasm

## Abstract

Background and objective

Nasopharyngeal carcinoma (NPC) presents a complex epidemiological pattern influenced by demographic characteristics, risk factors such as Epstein-Barr virus (EBV) infection, and smoking. Understanding the clinical profile and optimizing treatment strategies for NPC requires comprehensive analyses of these factors. In light of this, this study aimed to analyze the epidemiological patterns, histological characteristics, and treatment outcomes of NPC patients diagnosed and treated at a single center from 2016 to 2023.

Materials and methods

This retrospective study was conducted at Tawam Hospital in the United Arab Emirates (UAE), focusing on patients diagnosed with NPC. It involved the analysis of patient age distribution to identify epidemiological patterns, histological examination to classify NPC types according to WHO guidelines, and evaluation of treatment outcomes based on induction chemotherapy regimens and concurrent chemoradiotherapy protocols. The effectiveness of various chemotherapy combinations, particularly cisplatin and 5-fluorouracil (Cis+5FU), was assessed alongside the integration of advanced radiotherapy techniques like intensity-modulated radiotherapy (IMRT).

Results

In this study of 41 NPC patients, the age distribution varied widely, ranging from 10 to 74 years, with a mean age of >40 years. There was a significant male predominance (82.93%). Most patients were non-smokers (68.29%) and did not consume alcohol (92.68%), and there was a high prevalence of EBV positivity (100%). At diagnosis, 80.49% had no metastases. The primary treatment was chemotherapy induction, with a 73.17% uptake and a 92.68% completion rate, leading to a 65.85% complete response (CR) rate. No significant association was found between smoking status and treatment response (p=0.7657). Pathologically, non-keratinizing undifferentiated squamous carcinoma was the most common variant (75.61%). The Cis+5FU regimen was the most frequently employed method (56.67%), associated with a 76.47% CR rate. Concurrent chemotherapy was administered to 87.80% of patients, with the weekly Cis regimen being the most used one (56.09%), resulting in a significant CR rate. Combining radiation therapy with concurrent and induction chemotherapy yielded high CR rates (RT+cCT: 66.66%, RT+cCT+iCT: 80%). Survival analysis revealed the highest 36-month survival rate (46.43%) in the RT+cCT+iCT group, suggesting a potential benefit from incorporating induction chemotherapy into the treatment regimen.

Conclusions

This study illustrates the impact of demographic variables, EBV infection, and smoking on the development and treatment outcomes of NPC. It points to the success of customized chemotherapy and advanced radiotherapy strategies. Yet, it is limited by its retrospective nature and single-center focus, and hence we recommend multicentric studies to broaden the applicability of the results and improve NPC treatment approaches for varied patient groups.

## Introduction

Nasopharyngeal carcinoma (NPC), previously known as lymphoepithelioma, is a rare type of head and neck cancer originating from the nasopharynx's epithelial cells. The nasopharynx, an anatomically distinct component of the upper pharyngeal region, plays a pivotal role in the respiratory and auditory systems. Situated posterior to the nasal cavity, it encompasses several critical structures. The pharyngeal opening of the auditory tube, also known as the Eustachian tube, is instrumental in maintaining aural pressure equilibrium. Rosenmüller’s fossa lies adjacent to it, a notable recess posterior to the Eustachian tube opening, which is clinically significant due to its status as a predilection site for certain pathologies. The pharyngeal tonsil, or adenoids, located on the nasopharynx’s roof, is predominantly lymphatic and is a key site of immunological response, particularly in pediatric populations. The soft palate marks the boundary between the nasopharynx and the oropharynx, playing a critical role in speech and swallowing. Although not an intrinsic component of the nasopharynx, the nasal conchae significantly influence its function by conditioning air into the lungs. The pharyngeal fascia forms the superior aspect of the pharyngeal wall, integral to the structural integrity of the nasopharynx. The pharyngeal isthmus, a gateway between the nasopharynx and the remaining pharynx, is crucial for preventing nasal regurgitation during swallowing. Lastly, the posterior nasal apertures or choanae are the conduits connecting the nasal cavity to the nasopharynx, playing an integral role in respiratory flow [1,2].

NPC is distinct from other head and neck cancers in terms of its biology, epidemiology, histology, natural history, and response to treatment. NPC is associated with infection by the Epstein-Barr virus (EBV), a common herpesvirus that can cause infectious mononucleosis and other diseases. EBV is detected in almost all NPC cases, especially in the endemic regions of East and Southeast Asia, where NPC is highly prevalent. Other risk factors for NPC include genetic susceptibility, environmental exposures, dietary habits, and immune status [3,4]. A systematic review has highlighted the importance of understanding these patterns for developing targeted prevention strategies and researching the causes of NPC, especially in pediatric populations [5]. Studies on Chinese immigrants have shown a decrease in age-standardized incidence rates of NPC among those living outside China, indicating the impact of environmental or lifestyle changes on disease risk and suggesting the potential for modifiable prevention factors [6]. Conversely, an upward trend in NPC incidence rates reported in Iran, particularly among males, necessitates a reassessment of local risk factors and the development of region-specific intervention strategies to address the rising NPC burden [7].

In the United Arab Emirates (UAE), the cancer statistics for the year 2022 highlight the significant public health challenge posed by this disease. A total of 5,526 cancer cases were reported, with 2,283 associated deaths. In this context, NPC represents a relatively minor yet significant proportion, with 36 cases in total, accounting for 0.56% of the cancer incidence. The cumulative risk of developing NPC in the UAE population is estimated at 0.09%, highlighting its rare but still notable presence within the broader oncological landscape. The mortality impact of NPC is substantial, with 23 deaths attributed to this specific cancer type, representing 0.43% of all cancer-related deaths and a cumulative risk of 0.07% for mortality. Additionally, the five-year prevalence data for NPC in the UAE indicates that there are 134 individuals living with this diagnosis, translating into a rate of 0.6 per 100,000 population [8].

The etiology and pathogenesis of NPC in the UAE are not well understood, and further research is needed to elucidate the role of genetic, viral, environmental, and lifestyle factors in this population. Some possible hypotheses include the influence of EBV genetic variation, exposure to indoor air pollution from incense burning, the consumption of salted fish and preserved foods, and the interaction of multiple infections and immune responses. The prevention and early detection of NPC in UAE may benefit from developing and implementing effective screening strategies, such as serological testing for EBV antibodies, nasopharyngeal endoscopy, and molecular biomarkers. The treatment and management of NPC in the UAE may also be improved by adopting multidisciplinary and evidence-based approaches, such as radiotherapy, chemotherapy, immunotherapy, and targeted therapy. The objective of this study is to analyze the epidemiological patterns, histological characteristics, and treatment outcomes of NPC patients diagnosed and treated at a single center from 2016 to 2023.

This article is partly based on the dataset that was used in a previous article published with Cureus, which can be found at https://www.cureus.com/articles/210940-understanding-the-radiation-dose-variability-in-nasopharyngeal-cancer-an-organs-at-risk-approach#!/. It must be noted that though the dataset is partly the same (descriptive data), the objective, statistical results, discussion, and conclusion are completely different and this article does not repeat the results presented in the published article and has completely omitted the data on organs at risk data used in that article.

## Materials and methods

Study population

This retrospective cohort study involved a dataset comprising patients diagnosed with NPC from 2016 to 2023. The dataset included demographic information, clinical characteristics, treatment details, and follow-up outcomes for each patient.

Data collection

Patient data were collected from electronic medical records, encompassing age at diagnosis, gender, nationality, comorbidities (diabetes, hypertension, dyslipidemia), lifestyle factors (smoking, alcohol consumption), family history of cancer, EBV status, tumor pathology, treatment modalities, response to treatment, progression, and survival outcomes. The date of diagnosis, last follow-up, progression, and survival times were recorded to facilitate survival analysis.

Treatment modalities

Treatment modalities were classified into three main groups based on the treatment regimen: (1) radiation therapy (RT) alone: this group included patients treated exclusively with RT; (2) radiation therapy with concurrent chemotherapy (RT+cCT): this group comprised patients who received RT alongside cCT; (3) radiation therapy with concurrent and induction chemotherapy (RT+cCT+iCT): patients in this group received a comprehensive treatment regimen, including RT, cCT, and additional iCT before the commencement of concurrent treatments.

Statistical analysis

The study employed logistic regression to assess the association between treatment modalities and the likelihood of a complete treatment response. A simple survival graph was plotted to evaluate the impact of different treatment regimens on patient survival outcomes. Due to certain constraints, these analyses were manually approximated for illustrative purposes.

Response assessment

The Response Evaluation Criteria in Solid Tumors (RECIST) is a set of published rules that provide definitions as to when cancer patients improve ("respond"), stay the same ("stable"), or worsen ("progress") during treatments. Initially published in 2000 and updated to RECIST 1.1 in 2009, these criteria offer a standardized approach for assessing cancer response to treatment on imaging studies like CT scans, MRIs, and X-rays. RECIST criteria focus primarily on the change in the size of measurable lesions and pertain to the following outcomes: complete response (CR): the disappearance of all target lesions; partial response (PR): at least a 30% decrease in the sum of diameters of target lesions, taking as reference the baseline sum diameters; stable disease (SD): neither sufficient shrinkage to qualify as PR nor sufficient increase to qualify as progressive disease (PD), taking as reference the smallest sum diameters while being studied; and PD: at least a 20% increase in the sum of diameters of target lesions, taking as reference the smallest sum in the study (this includes the baseline sum if that is the smallest in the study). In addition to the relative increase of 20%, the sum must demonstrate an absolute increase of at least 5 mm. The appearance of one or more new lesions is also considered progressions. RECIST 1.1 introduced changes such as reducing the number of lesions to be measured, clarifying lymph node assessment, and including new lesion criteria. RECIST V1.1 was used as the criteria for the response assessment in this study [9].

Ethical considerations

The Tawam Human Research Ethics Committee reviewed and approved the study protocol, with the approval number MF2058-2023-969. All patient data were anonymized and complete confidentiality and privacy were ensured.

## Results

**Figure 1:**
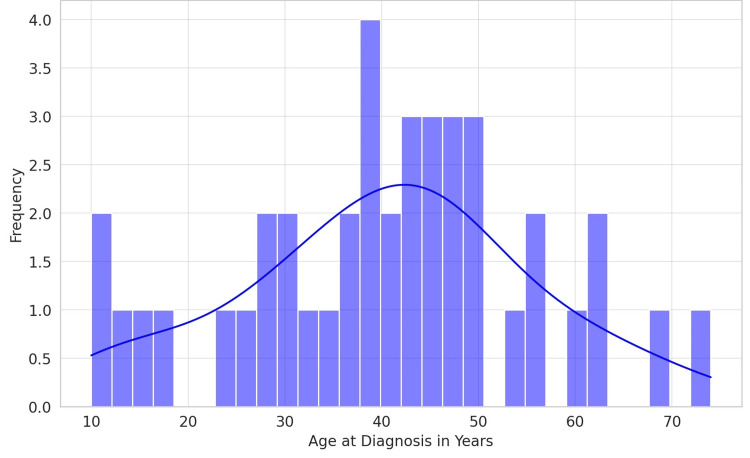
Age distribution in the cohort (N=41)

Data from the records of a total of 41 patients with NPC were collected. Age analysis revealed a broad age range among the patients, from very young individuals (10 years old) to elderly adults (74 years old), with a mean age of slightly over 40 years. This broad age distribution indicates that NPC affects a diverse age group, though the concentration towards middle age suggests a higher prevalence or detection rate in this demographic (Figure 1).

The majority of the patients diagnosed with NPC were male (34/41, 82.93%), indicating a higher prevalence in males compared to females (7/41, 17.07%). Regarding comorbidities such as diabetes and hypertension, most patients did not have these conditions, with 90.24% (37/41) reporting no diabetes and 82.93% (34/41) reporting no hypertension. An assessment of smoking habits among the patients showed that a significant number did not smoke (28/41, 68.29%), whereas 31.71% (13/41) were smokers. Alcohol consumption was relatively low among the patients, with only a small proportion admitting to alcohol use (3/41, 7.32%).

All patients in the dataset tested positive for EBV (41/41, 100%), highlighting its strong association with NPC. Regarding metastases, most patients (33/41, 80.49%) did not have metastases at the time of diagnosis, with only a small fraction (6/41, 14.63%) presenting with metastatic disease (five with bone and one with bone and liver). Treatment regimens included chemotherapy induction, with 73.17% of patients (30/41) undergoing this method, and a high treatment completion rate was observed (92.68%, 38/41). The response to treatment showed that 65.85% of patients (27/41) had a complete response, and when it came to disease progression, 63.41% of the cohort (26/41) were disease-free. Finally, the survival status indicated that 80.49% of the patients (33/41) were alive, with a mortality rate of 19.51% (8/41) within the dataset (Table 1).

**Table 1:** Demographic and clinical characteristics of nasopharyngeal cancer patients

Variable	Category	Count	Percentage
Gender	Male	34	82.93%
	Female	7	17.07%
Diabetes	No	37	90.24%
	Yes	4	9.76%
Hypertension	No	34	82.93%
	Yes	7	17.07%
Smoking	No	28	68.29%
	Yes	13	31.71%
Epstein-Barr virus (EBV)	Yes	41	100%
Metastases	No	33	80.49%
	Yes	6	14.63%
Chemotherapy induction	Yes	30	73.17%
	No	10	24.39%
Treatment completion	Completed	38	92.68%
Response to treatment	Complete	27	65.85%
Progression	Disease-free	26	63.41%
Status	Alive	33	80.49%
	Deceased	8	19.51%

**Figure 2:**
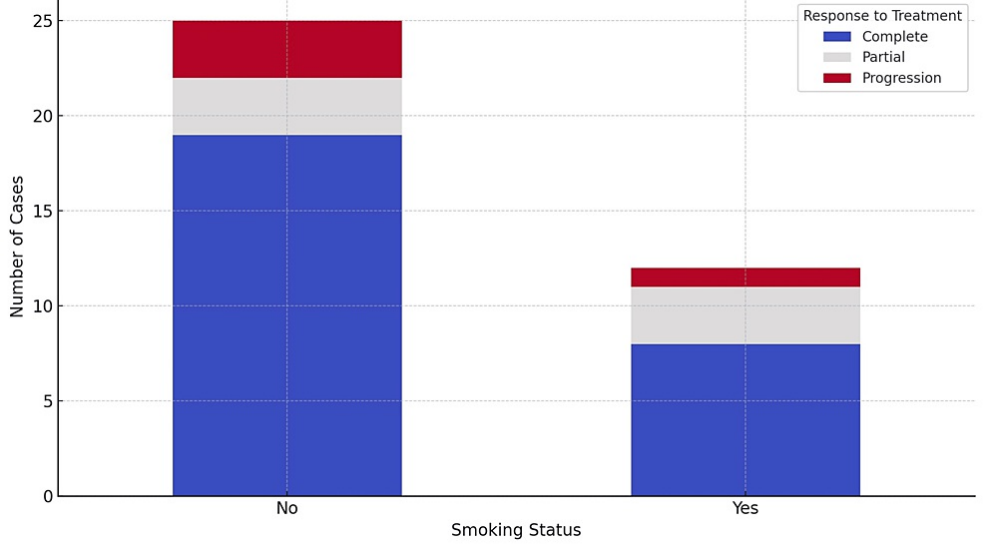
Smoking status and response to treatment

**Figure 3:**
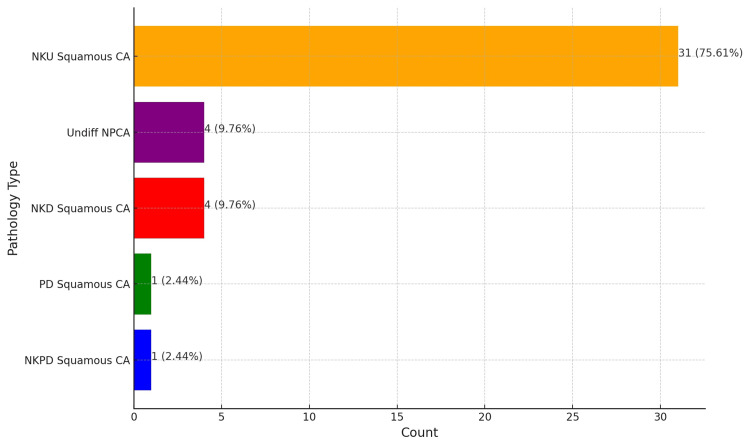
Distribution of pathology types in nasopharyngeal carcinoma NKU squamous CA: non-keratinizing undifferentiated squamous carcinoma; NKD squamous CA: non-keratinizing differentiated squamous cell carcinoma; Undiff NPCA: undifferentiated nasopharyngeal carcinoma; NKPD squamous CA: non-keratinizing poorly differentiated squamous carcinoma; PD squamous CA: poorly differentiated squamous cell carcinoma

To investigate the influence of smoking status on treatment response among NPC patients, a Chi-square test was performed. The analysis did not reveal a statistically significant association between smoking status and treatment outcomes, yielding a Chi-square value of 1.1472 with a p-value of 0.7657. To visually represent the relationship between smoking status and treatment response, a stacked bar chart was created, displaying the distribution of treatment responses within each smoking category. This visualization revealed that most of the treatment responses across smokers and non-smokers were categorized as "Complete", indicating a generally favorable outcome. However, the presence of 'Partial' and 'Progression' responses in both groups shows a variability in treatment efficacy that does not appear to be strongly influenced by smoking status (Figure 2).

our analysis of the distribution of pathology types revealed that non-keratinizing undifferentiated squamous carcinoma (NKU squamous CA) was the predominant pathology, representing 31 cases or 75.61% of the total. Non-keratinizing differentiated squamous cell carcinoma (NKD squamous CA) and undifferentiated NPC (Undiff. NPCA) accounted for four cases, equating to 9.76% of the dataset. Non-keratinizing poorly differentiated squamous carcinoma (NKPD squamous CA) and poorly differentiated squamous cell carcinoma (PD squamous CA) were identified in one case each, accounting for 2.44% of the cohort (Figure 3).

**Figure 4:**
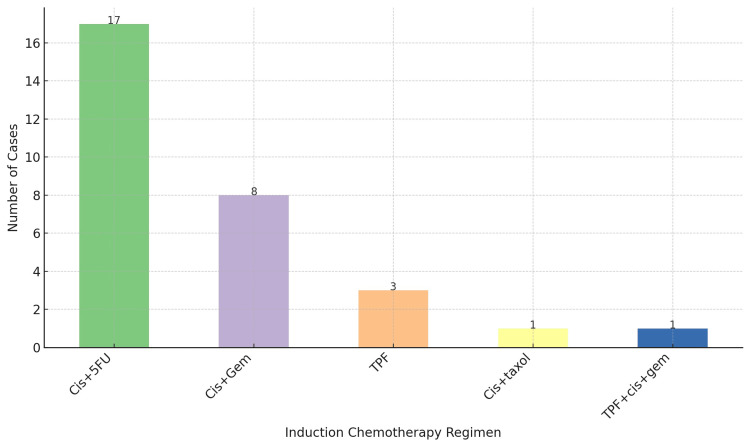
Induction chemotherapy regimen Cis+5FU: cisplatin and 5-fluorouracil; Cis+Gem: cisplatin and gemcitabine; TPF: docetaxel, cisplatin, and 5-fluorouracil; Cis+taxol: cisplatin and docetaxel; TPF+Cis+Gem: TPF followed by Cis+Gem

Based on our analysis of induction chemotherapy regimens, the most frequently used regimen was cisplatin and 5-fluorouracil (Cis+5FU), administered to 17 patients, representing 56.67% of the cases. The cisplatin and gemcitabine (Cis+Gem) regimen was employed in eight cases, accounting for 26.67%. The regimen consisting of cisplatin, 5-fluorouracil, and docetaxel (TPF) was chosen for three patients (10%), while cisplatin and docetaxel (Cis+taxol) were used in one case (3.33%). Additionally, a single case (3.33%) involved the use of a combination regimen, first receiving TPF and then Cis+Gem for the remaining cycles (TPF+Cis+Gem) (Figure 4). Almost all the patients received three cycles of induction therapy preceding radiotherapy.

**Figure 5:**
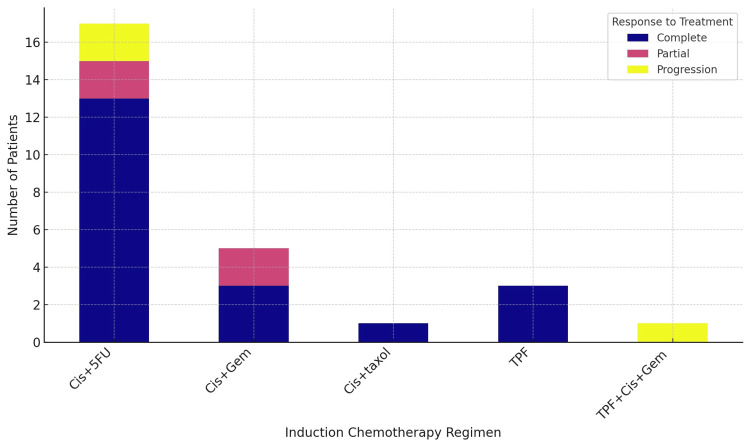
Induction regimen and disease response Cis+5FU: cisplatin and 5-fluorouracil; Cis+Gem: cisplatin and gemcitabine; TPF: docetaxel, cisplatin, and 5-fluorouracil; Cis+taxol: cisplatin and docetaxel; TPF+Cis+Gem: TPF followed by Cis+Gem

The Cis+5FU regimen resulted in a CR in 13 patients, PR in two patients, and progression in two patients. Since 17 patients were treated with this regimen, the CR rate was 76.47%, the PR rate was 11.76%, and the progression rate was 11.76%. Cis+Gem yielded a complete response in three patients and a partial response in two patients out of a total of eight patients treated, translating into a complete response rate of 37.5% and a partial response rate of 25%. No progression was recorded in patients receiving this regimen. Cis+taxol was administered to one patient, leading to a complete response (100% complete response rate). The TPF regimen achieved a complete response in all three patients treated, indicating a complete response rate of 100%. The TPF+cis+gem regiment was employed in one patient, which resulted in disease progression, marking a progression rate of 100% (Figure 5).

**Figure 6:**
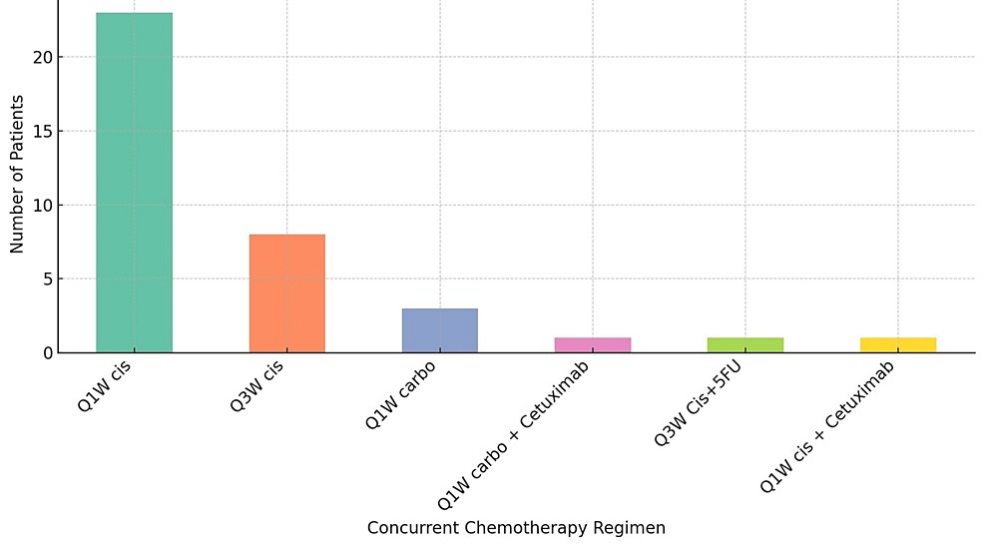
Concurrent chemotherapy regimen Q1W Cis: weekly cisplatin regimen; Q3W Cis: three-weekly cisplatin regimen; Q1W carbo: weekly carboplatin regimen; Q1W carbo + cetuximab: weekly carboplatin plus cetuximab regimen; Q3W Cis+5FU: three-weekly cisplatin plus 5-fluorouracil regimen; Q1W Cis + cetuximab: weekly cisplatin plus cetuximab regimen

At least 36 patients (87.80%) received concurrent chemotherapy, with weekly cisplatin regimen (Q1W Cis) being the most frequently used (n=23, 56.09%), followed by cisplatin every three weeks in eight patients (19.51%) and weekly carboplatin (Q1W carbo) in three patients (7.31%) (Figure 6). Cetuximab was used in two patients (4.87%).

**Figure 7:**
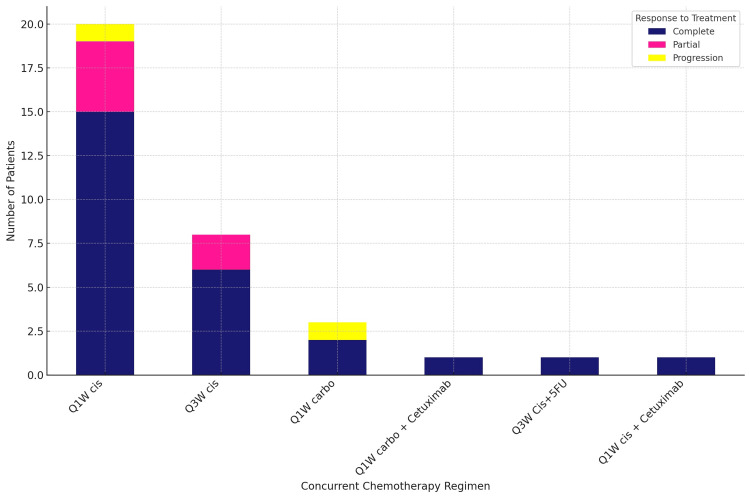
Concurrent chemotherapy and response to treatment Q1W Cis: weekly cisplatin regimen; Q3W Cis: three-weekly cisplatin regimen; Q1W carbo: weekly carboplatin regimen; Q1W carbo + cetuximab: weekly carboplatin plus cetuximab regimen; Q3W Cis+5FU: three-weekly cisplatin plus 5-fluorouracil regimen; Q1W Cis + cetuximab: weekly cisplatin plus cetuximab regimen

Of the 23 patients who received the Q1W Cis regimen, 15 (65.21%) had a complete response, four (17.39%) had a partial response, and one patient (4.34%) progressed. In the Q3W Cis group, out of the eight patients, six had a complete response, and two had a partial response (Figure 7).

**Figure 8:**
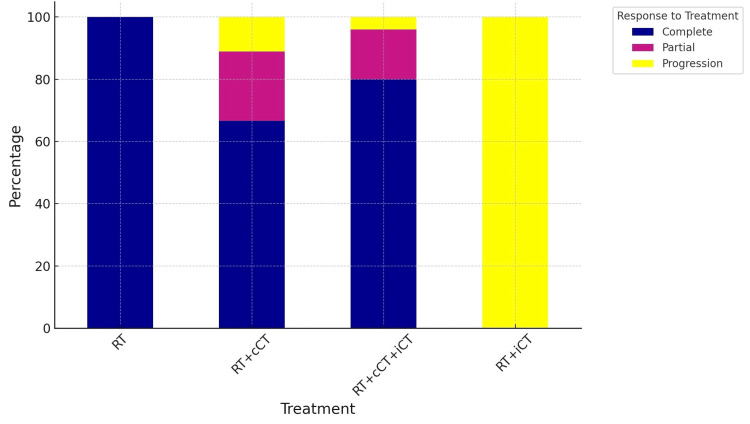
Response to different modalities RT: radiotherapy; RT+cCT: radiotherapy + concurrent chemotherapy; RT+cCT+iCT: induction chemotherapy followed by concurrent chemo-radiotherapy; RT+iCT: induction chemotherapy followed by radiotherapy

Nine patients who received RT+cCT demonstrated a diverse range of responses: six patients (66.66%) achieved a complete response, two patients (22.22%) exhibited a partial response, and one patient (11.11%) experienced disease progression. In 25 patients who underwent RT+cCT+iCT, the complete response rate improved to 80% (20 patients), with partial responses and progression observed in 16% (four patients) and 4% (one patient) respectively. Conversely, only one patient received RT+iCT and had a progression (Figure 8).

**Figure 9:**
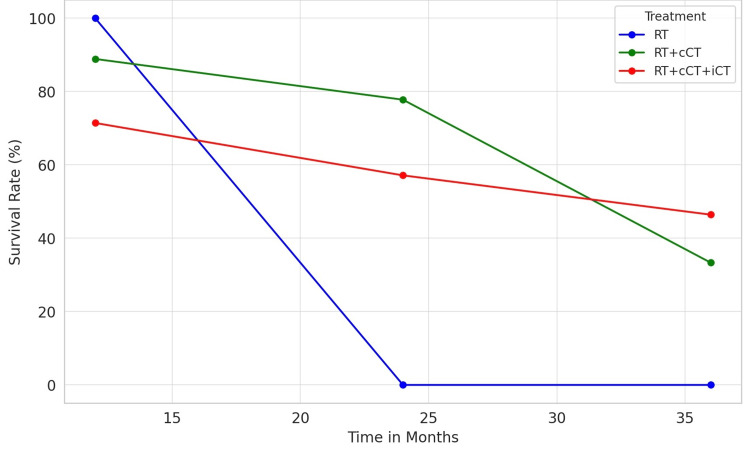
Survival over time for different treatment modalities RT: radiotherapy; RT+cCT: radiotherapy + concurrent chemotherapy; RT+cCT+iCT: induction chemotherapy followed by concurrent chemo-radiotherapy

A simple manual calculation of survival over time was performed for the three groups in the cohort: RT-only, RT+cCT, and RT+cCT+iCT (Figure 9). The RT-alone group is depicted in blue, showing a sharp decline from 100% survival at 12 months to 0% by 24 months; RT+cCT is shown in green, illustrating a gradual decrease in survival from 88.89% at 12 months to 33.33% at 36 months. RT+cCT+iCT, represented in red, starts with a 71.43% survival rate at 12 months and decreases to 46.43% at 36 months. The relatively higher survival rate at 36 months suggests a potential benefit of integrating iCT into the treatment regimen (Figure 9).

## Discussion

The age distribution of patients diagnosed with NPC in this dataset shows a broad range, suggesting that while NPC can occur across various age groups, there is a noticeable concentration among middle-aged individuals. This distribution may reflect underlying risk factors, demographic characteristics of the study population, or, potentially, the age-related likelihood of exposure to specific risk factors for NPC. This finding aligns with the literature, which identifies a differential age distribution of NPC in low-incidence regions compared to endemic regions. In low-incidence regions, the epidemiological pattern of NPC incidence demonstrates a bimodal distribution, with peaks observed in adolescent and young adult cohorts and a secondary increase in prevalence noted beyond the age of 65 years [10]. In stark contrast, endemic regions present a distinctly different profile, where the incidence of NPC begins to climb post-30 years of age, reaching a peak incidence within the 40-59 age group before experiencing a decline [11]. Moreover, a critical examination of age-specific survival rates reveals a prognostic disparity: individuals between 15 and 45 years manifest notably higher five-year survival rates than those between 65 and 74 years [12]. This disparity highlights the significant impact of age on survival outcomes in NPC, necessitating a better understanding of its epidemiological and clinical implications.

Histologically, NPC is predominantly classified into keratinizing squamous cell carcinoma, non-keratinizing carcinoma, and basaloid squamous cell carcinoma [13]. The non-keratinizing type is more closely associated with EBV infection and is more prevalent in endemic regions. The role of EBV in NPC pathogenesis is well-established, with viral DNA found in malignant cells, suggesting a direct oncogenic role [14]. Notably, plasma EBV DNA's inclusion in clinical guidelines is advocated, although the need for methodological harmonization is emphasized to ensure its global applicability [15]. Furthermore, the utility of EBV-specific cytotoxic T lymphocytes (CTLs) demonstrates the virus's potential as a target for adoptive immunotherapy. This approach has shown safety and effectiveness, particularly in post-transplant lymphoproliferative disorders, indicating a promising therapeutic avenue for EBV-associated malignancies [16]. In addition to being an indicator of response to treatment, EBV DNA has been found to have a significant role as a prognostic indicator of survival outcomes [17]. These findings highlight the multifaceted role of EBV in NPC, from diagnosis and prognosis to therapeutic intervention, emphasizing the importance of integrating EBV-specific biomarkers and targeted therapies into clinical practice. In this study, all the patients tested positive for the EBV pathogen.

Smoking has long been implicated in the etiology of various malignancies, including NPC. The relationship between smoking and NPC, however, is complex. While epidemiological studies have provided evidence that smoking increases the risk of NPC, the magnitude of this risk varies across different populations and is influenced by genetic, environmental, and viral factors, notably EBV infection [18]. The carcinogenic mechanisms by which smoking contributes to NPC development are multifaceted. Tobacco smoke contains numerous carcinogens that can cause DNA damage, leading to genetic mutations and chromosomal instability in nasopharyngeal epithelial cells. Additionally, smoking can impair immune function, potentially facilitating EBV infection or reactivation, which is a critical etiological factor in NPC [19]. The interaction between tobacco exposure and EBV's role in NPC suggests a synergistic effect, where smoking may exacerbate EBV's oncogenic potential or vice versa. Smoking has been associated with poorer outcomes in NPC patients. It can affect treatment efficacy, increase the likelihood of treatment-related toxicities, and adversely impact survival rates [20]. The exact mechanisms are not fully understood but may involve smoking-induced alterations in tumor biology, reduced treatment response, and increased risk of secondary malignancies.

WHO classification highlights three significant pathology variants of NPCs: non-keratinizing squamous cell carcinoma, keratinizing squamous cell carcinoma, and basaloid squamous cell carcinoma [21]. Non-keratinizing squamous cell carcinoma is further subdivided into differentiated and undifferentiated types. Undifferentiated variety is the most prevalent, particularly in endemic areas, and is associated with a better prognosis due to its higher responsiveness to radiotherapy and chemotherapy. In contrast, keratinizing squamous cell carcinoma, though less common, presents a different epidemiological profile and generally has a poorer response to radiation, necessitating more aggressive treatment approaches. Basaloid squamous cell carcinoma is recognized for its rarity and aggressiveness, characterized by distinctive histological features that indicate a more severe clinical course [22]. The profile of our study cohort aligns with the above classification, with close to 75% (31 patients) having non-keratinising undifferentiated squamous cell carcinoma.

Our findings highlight the pivotal role of induction chemotherapy in the management of NPC. The relatively higher survival rate at 36 months suggests a potential benefit of integrating induction chemotherapy into the treatment regimen. The predominant use of the Cis+5FU regimen, administered to 56.67% of the patients, demonstrates its significance in clinical practice. This regimen's high CR rate of 76.47% demonstrates its effectiveness in achieving tumor control before radiotherapy. This is consistent with previous findings indicating that Cis+5FU is a cornerstone in the treatment of NPC, offering a substantial benefit in tumor response [23]. The Cis+Gem regimen, although used less frequently, showed a complete response rate of 37.5%, which suggests that it may be a viable alternative for patients who are not candidates for the Cis+5FU regimen. The absence of disease progression in patients receiving the Cis+Gem regimen indicates its potential efficacy in controlling disease and improving patient outcomes [24]. The complete response rates observed with the Cis+taxol and the TPF regimens (100% in both cases) highlight the potential of these combinations in inducing significant tumor responses. However, their use in a smaller fraction of the patient population suggests a selective approach, possibly reserved for patients with specific clinical characteristics or for whom other regimens are deemed less suitable [25]. The unique case of the combined regimen (TPF for one cycle followed by two cycles cis+gem) resulting in disease progression underscores the complexity of treatment response and the need for individualized treatment planning. It suggests that while combination regimens can be effective, they may not be suitable for all patients, and their use should be carefully considered [26].

The high rate of concurrent chemotherapy administration (87.80%), with the majority receiving the Q1W Cis regimen, further emphasizes the role of chemotherapy in the comprehensive treatment of NPC. The complete response rate of 65.21% with the Q1W Cis regimen endorses its efficacy as a concurrent treatment alongside radiotherapy, aligning with findings highlighting the benefit of integrating chemotherapy with radiation therapy to enhance treatment outcomes [27]. The results of this study contribute to the growing body of evidence supporting the role of chemotherapy in the management of NPC. They underline the importance of selecting the appropriate chemotherapy regimen based on individual patient factors to optimize treatment outcomes.

All patients in our cohort received radiotherapy as it is a cornerstone in treating NPC, given the tumor's radiosensitive nature and anatomical location, which often precludes surgical intervention. The evolution of radiotherapy techniques from two-dimensional to intensity-modulated radiotherapy (IMRT) has significantly improved treatment outcomes by enhancing tumor control while minimizing toxicity to surrounding tissues [28]. The efficacy of radiotherapy as a primary treatment modality in NPC is well-documented, with high local control rates and improved survival outcomes. IMRT, in particular, has been a game-changer, offering the ability to deliver high-dose radiation to the tumor with precise targeting, thereby reducing the dose to critical structures such as the salivary glands, brainstem, and spinal cord [29]. One study has shown that IMRT leads to superior survival rates and reduced toxicities compared to older radiotherapy techniques, establishing it as the standard of care for NPC [30].

Concurrent chemoradiotherapy (CCRT) has further enhanced the therapeutic landscape of NPC, becoming the standard treatment for locally advanced disease. The addition of chemotherapy to radiotherapy has been shown to improve overall survival and disease-free survival and reduce distant metastases. This shows the synergistic effect of a multimodality approach in the management of NPC, where the integration of chemotherapy with radiotherapy has been proven to confer a significant survival advantage over radiotherapy alone [31]. The role of radiotherapy in the recurrent and metastatic setting also merits attention. For patients with locoregionally recurrent NPC, re-irradiation using IMRT has demonstrated promising results in terms of local control and survival, albeit with a higher risk of toxicity. This highlights the need for careful patient selection and optimization of radiotherapy plans to balance efficacy with potential adverse effects [32]. Advances in radiotherapy techniques continue to evolve, with emerging modalities such as robotic radiosurgery and proton therapy offering potential benefits in reducing radiation dose to non-target tissues and possibly further improving the therapeutic ratio [33-36].

Limitations

This study has a few limitations. Its single-center retrospective design means that the findings may not be generalizable to the broader population. Given the lower incidence of NPC in the UAE, the numbers of patients in the study dataset are considered substantial but still lack the power to render the findings generalizable to the whole region. Another drawback is that the majority of the patient cohort were expats, and a delay was observed in the starting of their treatments as these patients desire more often to travel to their home country to seek multiple opinions before returning to the UAE to begin treatment.

## Conclusions

This study provides a detailed examination of NPC, focusing on the age distribution of patients, histological classifications, and treatment outcomes. It highlights the variable incidence of NPC across different age groups, with a notable prevalence among middle-aged individuals, suggesting a complex interplay of demographic factors, EBV infection, and smoking in the disease's etiology and progression. The study highlights the role of EBV in NPC pathogenesis and advocates for targeted therapeutic interventions and integration of EBV-specific biomarkers in clinical practice. The efficacy of various induction chemotherapy regimens, alongside the significant role of concurrent chemoradiotherapy, underscores the importance of a multimodal approach in NPC management. The study affirms the benefits of integrating chemotherapy with advanced techniques like IMRT to enhance treatment outcomes.

Future research should focus on multicentric studies to validate these observations and refine NPC treatment protocols, aiming to achieve improved patient care across various populations.
